# WGX50 attenuates radiation enteritis by targeting ferroptosis and redox homeostasis via EGFR

**DOI:** 10.1186/s10020-025-01375-3

**Published:** 2025-10-08

**Authors:** Zhijing Yin, Guanjun Chen, Yunqing Liu, Yiqi Tan, Jingyi Tang, Ganghua Zhang, Dongqing Wei, Yuxing Zhu, Ke Cao

**Affiliations:** 1 Department of Oncology, Third Xiangya Hospital, Central South University, Changsha, China; 2 Department of Biology, University of North Carolina at Chapel Hill, Chapel Hill, NC USA; 3 School of Life Sciences and Biotechnology, Shanghai Jiao Tong University, Shanghai, China

**Keywords:** WGX50, Radiation enteritis, Oxidative stress, EGFR, Ferroptosis

## Abstract

**Background:**

Radiation enteritis (RE) is a common complication in patients undergoing abdominal and pelvic radiotherapy. Despite the advancements in radiotherapy, effective treatments remain limited. WGX50, a bioactive compound from Sichuan pepper, has shown anti-inflammatory and antioxidant properties. This study investigates the protective effects of WGX50 on RE, focusing on its potential to reduce radiation-induced damage in the intestine.

**Methods:**

Network pharmacology and molecular docking were used to identify the molecular targets of WGX50. In vitro, human intestinal epithelial cells (HIEC6) and colon cells (NCM460) were exposed to radiation and treated with WGX50. In vivo, C57BL/6 mice were administered WGX50 prior to radiation exposure. Various assays, including CCK-8, colony formation, flow cytometry, histopathology, and 16S rRNA sequencing, were performed to evaluate cell proliferation, apoptosis, oxidative stress, intestinal damage, and gut microbiota composition. Tissue transcriptome sequencing was conducted to explore differentially expressed genes.

**Results:**

In vitro, WGX50 significantly mitigated radiation-induced cell damage, enhanced cell proliferation, and reduced apoptosis at non-toxic concentrations. In vivo, WGX50 treatment preserved intestinal morphology and reduced inflammatory infiltration in irradiated mice. WGX50 also protected goblet cells, maintaining mucin production and epithelial barrier function critical for intestinal homeostasis. Molecular docking, dynamics simulations and surface plasmon resonance (SPR) revealed stable binding of WGX50 to Epidermal Growth Factor Receptor (EGFR), key targets involved in oxidative stress regulation and ferroptosis inhibition. Mechanistically, WGX50 upregulated the EGFR-SLC7A11-GPX4 axis, suppressing ferroptosis and protecting intestinal cells. Additionally, 16S rRNA sequencing showed that WGX50 mitigated radiation-induced gut microbiota dysbiosis, preserving microbial diversity and promoting beneficial bacterial populations.

**Conclusion:**

WGX50 demonstrates potent radioprotective effects by reducing oxidative stress, suppressing ferroptosis, and maintaining intestinal homeostasis, including goblet cell function and gut microbiota composition. These findings support WGX50’s potential as a novel therapeutic agent for the prevention and treatment of radiation enteritis.

**Supplementary Information:**

The online version contains supplementary material available at 10.1186/s10020-025-01375-3.

## Introduction

Radiation enteritis (RE) is a prevalent complication in patients undergoing radiotherapy for abdominal and pelvic malignancies. For instance, approximately 80% of cervical and endometrial cancer patients require radiotherapy (D'Auge et al. 2023). Despite advancements in intensity-modulated radiotherapy (IMRT), which allows precise dose distribution, over half of these patients still experience acute radiation-induced intestinal injuries, with 17.9% progressing to chronic conditions (McCaughan et al. 2021; Ahmed and Ahmed 2022). The risk is further elevated in patients who undergo total hysterectomy, as post-surgical displacement of intestinal loops into the pelvic cavity increases radiation exposure.

RE manifests as radiation-induced enteritis or colitis, depending on the affected site. The small intestine, being highly radiation-sensitive, often suffers significant toxic effects. Conversely, the relative fixed position of the colon, particularly the rectum, makes radiation proctitis more pronounced in later stages of treatment (Omer et al. 2022). In addition to its high incidence and significant impact on patients’ quality of life, another challenge of RE is the difficulty of treatment. Glucocorticoids combined with non-steroidal anti-inflammatory drugs (NSAIDs) can effectively alleviate symptoms, but their long-term use is limited by severe systemic side effects(Lian et al. 2023). Therefore, developing new therapeutic agents targeting the pathogenesis of RE to replace traditional treatment approaches is of great clinical significance.

The plant kingdom is a vast reservoir of chemically diverse and biologically active compounds. Herbal formulations, crude plant extracts, and even dietary components represent some of the most common sources of plant-derived phytochemicals used in cancer treatment. Numerous plant-based antioxidants, such as resveratrol, catechins, and curcumin, have been extensively studied for their potential as adjuvants in anti-cancer therapy (Cardoso et al. 2020; Ren et al. 2021; Shabbir et al. 2021; Esmeeta et al. 2022). Additionally, commonly consumed plants, including tea, Ganoderma, and chili peppers, have been shown to exhibit potent antioxidant properties (Yuan et al. 2023). Beyond their unique bioactivities—such as free radical scavenging, anti-inflammatory effects, and immune system modulation—plant extracts offer a significant advantage in terms of safety and tolerability, making them highly compatible with human physiology (Yuan et al. 2023; Hou et al. 2020).

WGX50 is a bioactive compound isolated from the pericarp of Sichuan pepper (*Zanthoxylum*). Our previous studies have demonstrated that WGX50 can directly reduce amyloid-β (Aβ) oligomers in the cerebral cortex or indirectly suppress Aβ-induced neuroinflammation by activating the JAK2/STAT3 and PI3K/AKT pathways, thereby alleviating Alzheimer’s disease (AD) (Tang et al. 2013; Shi et al. 2016; Hou et al. 2017). Furthermore, WGX50 has been found to inhibit ferroptosis in cardiomyocytes, effectively mitigating doxorubicin (DOX)-induced cardiotoxicity (Tai et al. 2023). These findings provide a strong theoretical basis for the potent anti-inflammatory and antioxidant properties of WGX50.

To investigate whether WGX50 confers a protective effect against RE, in this study, we first confirmed that WGX50 effectively protects both cells and the intestinal tract in mice from radiation-induced damage without exhibiting significant toxicity. Subsequently, through a series of in vitro and in vivo experiments, we further validated the radioprotective effects of WGX50 on the intestine. Finally, leveraging transcriptomic sequencing, we identified potential therapeutic targets and elucidated the underlying molecular mechanisms, providing new strategies and theoretical insights for the prevention and treatment of RE.

## Methods

### Identification of drug targets and disease-associated targets

The chemical structure of WGX50 was obtained from previous literature (Fan et al. 2015), and potential molecular targets were predicted using SwissTargetPrediction (http://swisstargetprediction.ch/). Targets were screened based on a probability threshold of > 0.1. Disease-associated targets for radiation enteritis were retrieved from GeneCards, DrugBank, and the Gene database. In GeneCards, genes were filtered using a relevance score threshold of > 5.

### PPI network construction and core module analysis

The overlapping drug-disease targets were imported into the STRING database (https://cn.string-db.org/) to construct a protein–protein interaction (PPI) network. STRING-derived interaction pairs (A-B, B-A) were subsequently imported into Cytoscape, and node degrees were calculated using CytoNCA. The MCODE algorithm was employed to identify key regulatory modules, with the degree cutoff set to 2 to extract two high-score modules. Further analysis within these modules was performed using cytoHubba, where Hubba genes were ranked using the Maximal Clique Centrality (MCC) algorithm. The top 10 hub genes were selected and visualized for further investigation.

### Cell, animal, and reagent sources

Human intestinal epithelial cells HIEC6 and colon epithelial cells NCM460 were purchased from Wuhan Procell Life Technology (Wuhan, China). Both cell lines were cultured in RPMI-1640 medium, supplemented with 10% fetal bovine serum and 1% penicillin–streptomycin. The cells were incubated in a constant temperature incubator at 37 °C with 5% CO_2_. C57BL/6 mice, 6 weeks old and weighing an average of 20 g, were purchased from Hunan SJA Laboratory Animal Co., Ltd [License No. SCXK(Hunan) 2019–0004]. The mice were housed in the SPF-grade laboratory of the Experimental Animal Department, Central South University [License No. SYXK(Hunan) 2020–0019]. The WGX50 powder was provided by Professor Wei Dongqing’s research team at Shanghai Jiao Tong University.

### Radiation treatment

All cell and animal irradiation was performed using the Faxitron MultiRad 225 small animal irradiator (USA). The X-ray tube voltage was set within the range of 180-225kV, with a current of 20mA, and the source-to-skin distance (SSD) was 50 cm. For cellular experiments, a single 6 Gy dose was selected for CCK-8, flow cytometry and other assays based on preliminary work demonstrating optimal phenotypic responses at 60–70% confluence, while colony formation assays used lower gradient doses (0/2/4/6 Gy) due to the extreme radiosensitivity of sparsely seeded cells, where 6 Gy proved almost lethal. The dose rate was maintained at 300 cGy/min throughout cellular irradiation to ensure experimental consistency. To establish a partial-body irradiation (PBI) model while protecting vital organs, anesthetized C57BL/6 mice (0.3% pentobarbital sodium, i.p.) were positioned prone and immobilized with transparent tape. A customized 3-mm lead Shield was used to precisely cover the head, thorax, pelvis, and limbs, creating a defined abdominal irradiation field measuring approximately 2.5 cm × 1.5 cm (length × width), which extended from the xiphoid process to the upper anal margin. Radiation was delivered at a dose rate of 150 cGy/min to achieve a total dose of 13 Gy.

### CCK-8 assay

Seed the cells at a density of 2,000 cells per well in a 96-well plate with 100 μL of complete culture medium. After pre-treating the cells in each group with different concentrations of WGX50 for 24 h, the irradiation group received a total dose of 6 Gy. All cells will then continue to be cultured for 24, 48, 72, and 96 h. After the designated time periods, add 10 μL of CCK-8 reagent (Beyotime, China) to each well and incubate for 2 h. The absorbance at 450 nm will be measured with a microplate reader (ThermoFisher Scientific, USA) to assess the proliferation capacity of the cells in each group.

### Colony formation assay

Cells were seeded at a density of 800 cells per well in six-well plates. After the cells adhered to the surface, the treatment group was incubated with 20 μM WGX50 for 72 h. Following treatment, the cells in the irradiation group were exposed to different doses of radiation and cultured for 14 days. During the culture period, the medium was changed every three days, and cell conditions were monitored. After two weeks, when the majority of individual colonies contained more than 50 cells, the cells were fixed with 500 μl of 4% paraformaldehyde for 30 min and washed. Subsequently, 500 μl of 1% crystal violet staining solution was added to each well for 20 min. After multiple washes with PBS, the plates were air-dried in a laminar flow hood. The number of cell colonies in each well was photographed and recorded.

### Immunofluorescence

Reactive oxygen species (ROS) levels in cells from different groups were quantified using a ROS detection kit (Beyotime, China). The DCFH-DA fluorescent probe was diluted to 10 μM in culture medium at a 1:1000 ratio and incubated at 37 °C for 20 min. The cells were then washed three times with serum-free medium to remove any unbound probe. Fluorescence intensity was measured using a laser scanning confocal microscope with an excitation wavelength of 488 nm and an emission wavelength of 525 nm, and fluorescence was monitored at each time point.

### Flow cytometric analysis

To investigate the effects of ionizing radiation and WGX50 on oxidative stress and apoptosis levels in HIEC6 and NCM460 cells, we performed the following analyses using flow cytometry:

ROS Fluorescence Flow Cytometry: The total intracellular ROS levels were measured using the CellROX™ Green probe (ThermoFisher Scientific, USA). Cells from each group were incubated with 5 μM probe in a 37 °C, 5% CO_2_ incubator for 30 min to ensure sufficient probe entry and oxidation by ROS. After incubation, cells were washed three times with pre-chilled PBS to remove any unbound probe. Fluorescence intensity was then measured using a flow cytometer in the FITC channel, with changes in fluorescence intensity reflecting the levels of ROS in the cells.

Cell Apoptosis Flow Cytometry: Cell apoptosis levels were assessed using the Annexin V-FITC/PI dual-staining method. First, cells were digested with 0.25% trypsin, collected by centrifugation, and washed once with pre-chilled PBS. The cells were then resuspended in 500 μL PBS and stained with 5 μL of Annexin V-FITC and 10 μL of propidium iodide (PI) staining solution (Beyotime, China) for 20 min in the dark at room temperature. After the staining reaction, cells were immediately analyzed by flow cytometry. Annexin V-FITC was detected in the FITC channel, and PI was detected in the PE channel to differentiate early apoptotic cells (Annexin V-FITC positive, PI negative) from late apoptotic or necrotic cells (Annexin V-FITC positive, PI positive/PI alone positive).

Mitochondrial Membrane Potential Detection: Mitochondrial membrane potential changes were assessed using the JC-1 staining method. After collecting the cell pellet, 1 mL of JC-1 staining working solution (Beyotime, China) was added, and the mixture was gently vortexed to ensure even distribution of the dye. The cells were incubated for 20 min to allow JC-1 to enter the mitochondria. After incubation, the cells were washed twice with PBS to remove unbound dye and then resuspended in 1 mL PBS. Flow cytometry was performed, with JC-1 monomers detected in the FITC channel and JC-1 aggregates detected in the PE channel. A decrease in mitochondrial membrane potential is indicated by an increase in the JC-1 monomer ratio and a decrease in the polymer ratio.

FlowJo (Version 10.7.2) software was used to analyze the experimental data. The intracellular ROS levels were quantified by changes in fluorescence intensity in the FITC single channel, with an increase in fluorescence intensity indicating heightened oxidative stress levels. Apoptotic cells were distinguished by fluorescence signals from Annexin V-FITC and PI, and the proportion of apoptotic cells was calculated. Additionally, fluorescence intensity changes in JC-1 monomers and aggregates were analyzed to evaluate mitochondrial membrane potential changes. All experiments included control groups to ensure data reliability and reproducibility.

### Western blotting

Total protein was extracted from NCM460 cells, and protein concentration was measured using the BCA assay. Equal amounts of protein samples were mixed with sample loading buffer, boiled for denaturation, and then separated by SDS-PAGE gel electrophoresis (Servicebio, China) using a standardized 20 μg loading volume across all samples. After electrophoresis, the proteins were transferred to a PVDF membrane and blocked with 5% non-fat milk at room temperature for one Hour. The membrane was then incubated overnight at 4 °C with primary antibodies, including GAPDH (1:10,000, Abcam, UK), SLC7A11 (1:1,000, Zenbio, China), GPX4 (1:10,000, Huabio, China), EGFR (1:5000, abmart, China), p-EGFR (1:1000, abmart, China), and β-actin (1:10,000, Abcam, UK). The following day, the membrane was washed three times with TBST for 10 min each time, and then incubated at room temperature for one hour with HRP conjugated goat anti-rabbit IgG secondary antibody (1:10,000, Abcam, UK). After further washing, chemiluminescent detection was performed using an ECL substrate, and images were captured with a gel imaging system. GAPDH was used as an internal control to quantify the relative expression levels of SLC7A11 and GPX4. β-actin was used as an internal control to quantify the relative expression levels of EGFR and p-EGFR.

### Animal experiment

After allowing all mice to acclimate to their environment for one week, an initial experiment was conducted to assess the toxicity of WGX50. Mice were randomly divided into two groups (*n* = 5): the saline treatment group (Control) and the WGX50 treatment group (WGX50). For four weeks, these mice were orally gavaged with 200 μL of WGX50 solution (28 mg/kg/day) or an equal volume of saline (Tang et al. 2013). The general condition and body weight of the mice were monitored daily. After confirming that WGX50 did not induce toxicity, a subsequent experiment was conducted to evaluate its protective effects against irradiation.

For this second experiment, mice were randomly divided into three groups (*n* = 5): the saline treatment group (Control), the saline treatment + irradiation group (IR + NS), and the WGX50 treatment + irradiation group (IR + WGX50). Mice in the IR + NS and IR + WGX50 groups received oral gavage of either saline or WGX50 (28 mg/kg/day) for three weeks prior to irradiation. Following this, the IR + NS and IR + WGX50 groups were subjected to a single 13 Gy dose of whole-abdominal irradiation. Seven days post-irradiation, the irradiated groups exhibited significant deterioration in general condition, with weight loss reaching up to 20% of their pre-irradiation body weight. All mice were euthanized by cervical dislocation, and the colon, small intestine, and other organs were collected for subsequent analysis. To ensure consistency in phenotypic analysis, three representative samples per group with minimal inter-individual variability were randomly selected for downstream analyses and statistical evaluation. Additionally, to further assess potential dose-dependent effects and long-term safety, a separate cohort of mice received WGX50 at 28 mg/kg or 56 mg/kg via gavage for six weeks. Multiple indicators, including behavioral parameters, hematological and biochemical profiles, organ morphology, and histopathology, were evaluated.

### Histological analyses

Portions of the small intestine and colon, as well as other organ tissues, were fixed in neutral formalin at room temperature for 24 h, followed by paraffin embedding. The paraffin-embedded tissue sections were baked at 60 °C for 12 h and then dewaxed: the sections were immersed in xylene for 20 min, repeated three times; followed by dehydration in graded ethanol solutions (100%, 95%, 85%, and 75%) for 5 min each; and finally washed in distilled water for 5 min.

The H&E staining procedure was as follows: the sections were stained in hematoxylin for 10 min, washed in distilled water, and then blued by briefly immersing them in an ammonia water solution to differentiate the color. Subsequently, the sections were stained in eosin for 5 min, washed in distilled water, and then dehydrated through graded ethanol solutions (75%, 85%, 95%, and 100%) for 5 min each. Afterward, the sections were cleared in xylene for 10 min, dried, and mounted with neutral gum. The sections were then observed under a microscope for morphological changes. Crypt survival was quantified in H&E-stained sections by counting intact crypts containing ≥ 10 basophilic epithelial cells with distinct mitotic figures per intestinal circumference (Lee et al. 2019).

To assess goblet cells and mucin secretion in the intestine, PAS staining was performed. After dewaxing and rehydration, sections were oxidized in 0.5% periodic acid solution for 10 min, washed in distilled water, and stained with Schiff's reagent for 15 min. The sections were then washed under running water for 10 min, counterstained with hematoxylin for one minute, washed with distilled water, dehydrated, cleared, and mounted. PAS-positive areas appeared magenta and were used to evaluate goblet cell numbers and mucin secretion levels.

For the quantitative detection of inflammatory markers and target protein expression in tissues, tissue sections were processed through baking, dewaxing, and rehydration, followed by blocking endogenous peroxidase activity with 3% hydrogen peroxide at 25 °C for 25 min. The sections were then incubated overnight at 4 °C with primary antibodies, including iNOS (1:100, Zenbio, China), Ki67 (1:200, Abiowell, China), EGFR (1:200, Abiowell, China), SLC7A11 (1:200, Abiowell, China), GPX4 (1:200, Abiowell, China), CLCA1 (1:200, Abiowell, China), and MUC2 (1:200, Abiowell, China). On the following day, the sections were incubated at room temperature with anti-rabbit IgG-HRP secondary antibody for 90 min. After DAB color development, the sections were counterstained with hematoxylin, dehydrated, cleared, and mounted. Positive signals appeared brown and were used to evaluate the expression levels of the target proteins.

All stained sections were scanned using a panoramic scanner (Pannoramic MIDI II-3D HISTECH Ltd.) and images were captured using SlideViewer (Version 2.5) at the same magnification. ImageJ (Java 1.8.0_322) software was used for quantitative analysis of the positive areas in HE, PAS, and immunohistochemical staining, including the statistical analysis of positive areas or positive cell numbers.

### Hematological, biochemical, and ELISA assays

Blood samples from all experimental groups were collected via the orbital venous plexus and used for hematological and biochemical analyses. Hematological analysis was performed using an automated hematology analyzer to measure white blood cell count (WBC), red blood cell count (RBC), hemoglobin concentration (HB), platelet count (PLT), and other relevant parameters. Biochemical analysis was conducted using an automated biochemical analyzer to determine the levels of aspartate aminotransferase (AST), alanine aminotransferase (ALT), lactate dehydrogenase (LDH), blood urea nitrogen (BUN), creatinine (CREA), creatine kinase (CK), and cardiac troponin T (CTnT), which were used to assess liver and kidney function as well as metabolic status.

Inflammatory factors and peroxide levels were measured using ELISA. Colon tissue samples were homogenized and diluted to the same concentration. The homogenates were then added to pre-coated ELISA plates specific for inflammatory factors (IL-4, IL-6, TGF-β, etc.) and peroxides (H2O2, MDA, NO, etc.), following the instructions provided in the ELISA kits (Enzyme-linked Biotechnology, China). After incubation, HRP-conjugated secondary antibodies were added, and after TMB substrate development, absorbance at 450 nm was measured using a microplate reader. The concentrations of each marker were calculated using standard curves.

### 16S rRNA gut microbiota profiling

After collecting intestinal samples, fecal samples from all mice in the three experimental groups (n = 5 per group; total n = 15) were immediately frozen using dry ice and stored at −80 °C for further analysis. Genomic DNA was extracted from the fecal samples using the DNA extraction kit D4015 (Omega Bio-Tek, Norcross, GA, USA). The 16S rRNA gene was amplified using forward primer 338 F (5'-ACTCCTACGGGAGGCAGCA-3') and reverse primer 806R (5'-GGACTACHVGGGTWTCTAAT-3') to target the V3-V4 region of the gene. The purified PCR amplicons were used for library construction with the TruSeq® DNA PCR-Free Sample Preparation Kit (FC-121–3001, Illumina, USA). Sequencing was then performed on the Illumina NovaSeq 6000 platform. The microbiome bioinformatics analysis was carried out under the guidance of Shanghai OE Biotech Co., Ltd. (China).

### Transcriptomic sequencing

Total RNA was extracted from both large and small intestine tissues. After confirming the RNA purity and integrity, the total RNA was reverse transcribed into complementary DNA (cDNA) using reverse transcriptase. The cDNA library was then constructed using high-throughput sequencing technology and amplified via polymerase chain reaction (PCR), with library concentration and distribution assessed. The library was subsequently loaded onto a sequencer for high-throughput sequencing to obtain extensive sequence data. Sequencing was performed on the Illumina NovaSeq 6000 platform at Shanghai OE Biotech Co., Ltd. (China), followed by subsequent bioinformatics analysis.

### Molecular docking

To investigate the binding and interaction patterns between WGX50 and its target proteins, human EGFR and CASP3 were selected as receptor proteins. The 3D structures of the proteins were obtained from the AlphaFold website. The 2D structure of WGX50 was retrieved from PubChem (http://pubchem.ncbi.nlm.nih.gov), and its 3D structure was generated using ChemOffice 20.0 software, then saved as a mol2 file. The protein targets with high-resolution crystal structures were selected from the RCSB PDB database (http://www.rcsb.org/), and the structures were processed using PyMOL2.6.0 software to remove water molecules, phosphate groups, and other irrelevant components, saving the files as PDB format. We used the Molecular Operating Environment (MOE) 2019 software to minimize the compound’s energy, preprocess the target proteins, and identify potential active pockets. Finally, molecular docking was performed using MOE 2019 with 50 iterations. The binding activity of the compound and protein was evaluated based on the binding energy, and the results were visualized using PyMOL2.6.0 and Discovery Studio 2019 software.

### Protein–protein docking analysis

In this study, protein–protein docking analysis was performed using GRAMM, which is a rigid docking method where the conformations of both the ligand and receptor remain fixed during the docking process, with the optimal binding site identified on the protein surface. First, the protein sequences of EGFR and SLC7A11 were retrieved from the UniProtKB database. These sequences were then submitted to SWISS-MODEL to generate high-quality 3D protein structures. Finally, the resulting PDB files were used for protein–protein docking analysis.

### Molecular dynamics simulations

Molecular dynamics simulations of the CASP3-WGX50 and EGFR-WGX50 complexes were conducted using GROMACS 2020.3 software. The simulation box size was optimized to ensure that the distance between protein atoms and the box walls was greater than 1.0 nm. The system energy was minimized to reduce atomic overlap or clashes. NVT and NPT ensemble simulations were performed for equilibration, with a total simulation time of 100 ns. Temperature and pressure were controlled using the V-rescale and Parrinello-Rahman methods. Van der Waals interactions were calculated using the Lennard–Jones potential, with a nonbonded cutoff distance of 1.4 nm. The bond lengths of all atoms were constrained using the LINCS algorithm. Electrostatic interactions were calculated using the Particle Mesh Ewald (PME) method, with a Fourier spacing of 0.16 nm.

### Surface plasmon resonance (SPR) analysis

To evaluate the binding affinity between WGX50 and EGFR, SPR analysis was conducted using a Biacore 8 K instrument (GE Healthcare). Human EGFR protein (89.1 kDa) was immobilized on a CM5 sensor chip (Cytiva) via standard amine coupling chemistry. The coupling buffer used was 1 × PBS-P + (pH 7.4). After activation with EDC/NHS, EGFR was diluted to 50 μg/mL in acetate buffer and immobilized at a flow rate of 10 μL/min. The chip was then blocked with ethanolamine. A reference channel was prepared with the same procedure but without EGFR protein.

WGX50 was diluted in 1 × PBS-P + containing 5% DMSO and injected over the chip surface at a flow rate of 30 μL/min in a series of increasing concentrations. Each sample was flowed for 150 s, followed by regeneration with 10 mM glycine–HCl (pH 2.0). The binding kinetics were analyzed using Biacore Insight Evaluation Software and fitted to a 1:1 Langmuir binding model to calculate association (K_a_), dissociation (K_d_), and equilibrium dissociation constants (K_D_).

### Statistical analysis

In this study, bioinformatics analyses were performed using R (version 4.2.1), with Perl utilized for batch processing and data cleaning. Differential analysis was primarily conducted using the "limma" R package unless otherwise noted. For other group comparisons, the non-parametric Wilcoxon test was applied for two groups, while the Kruskal–Wallis test was used for three or more groups. Molecular docking was performed using Python (version 3.9.9). Experimental data were statistically analyzed using GraphPad Prism (version 9.0). All statistical tests were two-tailed, and significance was set at p < 0.05.

## Results

### Network pharmacology and molecular docking reveal the potential application and key targets of WGX50 in the treatment of RE

**Fig. 1:**
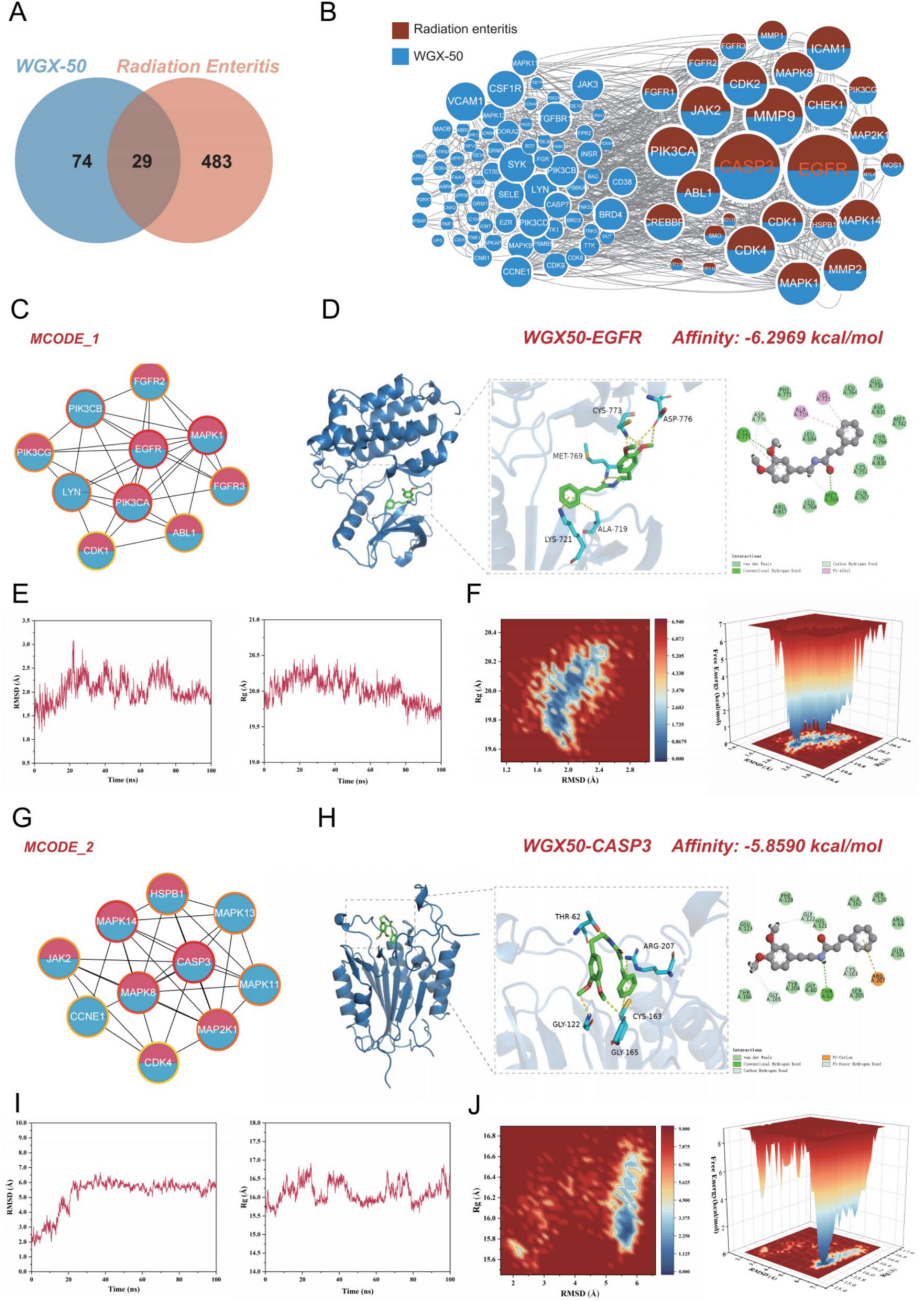
Potential mechanisms and key targets of WGX50 in the treatment of RE based on network pharmacology and molecular docking. **A** Intersection analysis of WGX50 and RE target proteins. **B** Predicted potential targets of WGX50 for RE treatment: Blue nodes represent WGX50 targets, red nodes represent RE targets, and bicolor nodes indicate shared targets; node size is proportional to the degree value. **C** PPI network centered around EGFR,where a darker outer ring color represents a tighter binding between the protein and EGFR. **D**) Molecular docking visualization of WGX50 and EGFR. **E** Molecular dynamics simulation of the EGFR-WGX50 complex showing the RMSD and Rg curves. **F** 3D and 2D representations of the free energy landscape from molecular dynamics simulations of the EGFR-WGX50 complex. **G** PPI network centered around CASP3, where a darker outer ring color represents a tighter binding between the protein and CASP3. **H** Molecular docking visualization of WGX50 and CASP3. **I** Molecular dynamics simulation of the CASP3-WGX50 complex showing the RMSD and Rg curves. **J** 3D and 2D representations of the free energy landscape from molecular dynamics simulations of the CASP3-WGX50 complex

Based on the chemical structure of WGX50, potential molecular targets were identified using SwissTargetPrediction with a screening criterion of Probability > 0.1, resulting in a total of 103 targets. A total of 5,140 publications were identified by searching the keyword 'radiation enteritis', and after filtering for a relevance score > 5, a total of 491 disease-associated genes were obtained. Additionally, 16 genes were retrieved from DrugBank and 21 from the Gene database. After merging and deduplicating data from GeneCards, DrugBank, and Gene, 512 unique disease-associated genes were identified. By intersecting the predicted WGX50 targets with disease-associated genes, 29 overlapping genes were identified (Fig. 1A). To elucidate the relationship between WGX50 and radiation enteritis, a drug-target and disease-target interaction network was constructed using Cytoscape, in which blue nodes represent drug targets and red nodes denote disease-associated genes (Fig. 1B). The STRING-derived interaction pairs (A-B, B-A) were imported into Cytoscape, and node degrees were calculated using CytoNCA. Node sizes were adjusted based on degree values, highlighting Epidermal Growth Factor Receptor (EGFR) and Cysteine-dependent aspartate-specific protease-3 (CASP3) as the top-ranked nodes with degrees of 538 and 488, respectively. Further network analysis using MCODE and cytoHubba revealed two key functional modules: MCODE_1 (score = 7.867) with EGFR as the hub (Table 1), and MCODE_2 (score = 6.556) with CASP3 as the hub (Table 2). These findings highlight EGFR and CASP3 as pivotal regulators in the drug-disease interaction network (Fig. 1C, G). To further investigate the interaction between the small molecule active compound WGX50 and the protein targets EGFR and CASP3, molecular docking was performed. Generally, docking energy values of < −4.25 kcal/mol indicate some binding activity, < −5.0 kcal/mol suggest good binding activity, and < −7.0 kcal/mol represent strong binding activity(Paggi et al. 2024). The docking results showed that the Cys773 and Met769 residues of the EGFR protein receptor form hydrogen bonds with WGX50, while the Ala719, Lys721, and Cys773 residues form hydrophobic interactions with WGX50. Additionally, Asp776 and Met769 form van der Waals interactions with WGX50. The molecular docking energy was −6.2969 kcal/mol (Fig. 1D). Furthermore, the Thr62 residue of the CASP3 protein receptor forms a hydrogen bond with WGX50, while Gly122, Gly165, and Cys163 residues form van der Waals interactions with WGX50. Additionally, Arg207 forms electrostatic interactions with WGX50. The molecular docking energy was −5.8590 kcal/mol (Fig. 1H). These results suggest that WGX50 exhibits good binding activity with both EGFR and CASP3 targets.

**Table 1:** Top 10 in network: Cluster 1 (Score: 7.867) ranked by MCC method

Rank	Name	Score
1	EGFR	474
2	MAPK1	366
3	PIK3CA	312
4	LYN	246
5	PIK3CB	240
6	ABL1	144
7	CDK1	126
8	FGFR3	120
9	FGFR2	120
10	PIK3CG	120

**Table 2:** Top 10 in network: Cluster 2 (Score: 6.556) ranked by MCC method

Rank	Name	Score
1	CASP3	780
2	MAPK14	744
3	MAPK8	742
4	MAP2K1	738
5	MAPK13	720
5	HSPB1	720
5	MAPK11	720
8	JAK2	50
9	CCNE1	26
10	CDK4	24

Given the favorable docking between WGX50 and CASP3/EGFR protein targets, molecular dynamics simulations were performed over 100 ns to further evaluate the interaction between the compound and protein targets. Root mean square deviation (RMSD) was chosen as a standard to assess the stability of the compound-protein binding system. During the simulation, both CASP3 and EGFR proteins reached equilibrium around 30 ns and 40 ns, respectively. The average RMSD values for the WGX50-CASP3 and WGX50-EGFR complexes were 5.22 Å and 2.05 Å, respectively, indicating that the complex systems formed between the compound and proteins may possess relatively high stability during the simulation. The radius of gyration (Rg) was used to demonstrate the compactness of the overall structure during the simulation process. It measures the distance between the center of mass and the terminal atoms over specific time intervals. The fluctuation ranges of the Rg values for CASP3 and EGFR during the simulation were 15.47–16.92 Å and 19.53–20.51 Å, with average Rg values of 16.14 Å and 20.02 Å, respectively. Overall, the compound WGX50 formed relatively stable complex systems with both CASP3 and EGFR during molecular dynamics simulations (Fig. 1E, I). In the free energy landscape of WGX50 binding with CASP3 and EGFR, multiple low-energy clusters were observed (Fig. 1F, J), which likely explain the high binding affinity and stability of WGX50 with the CASP3 and EGFR receptors.

### In vitro and in vivo evaluation of the effective concentration and safety of WGX50 for RE treatment

**Fig. 2:**
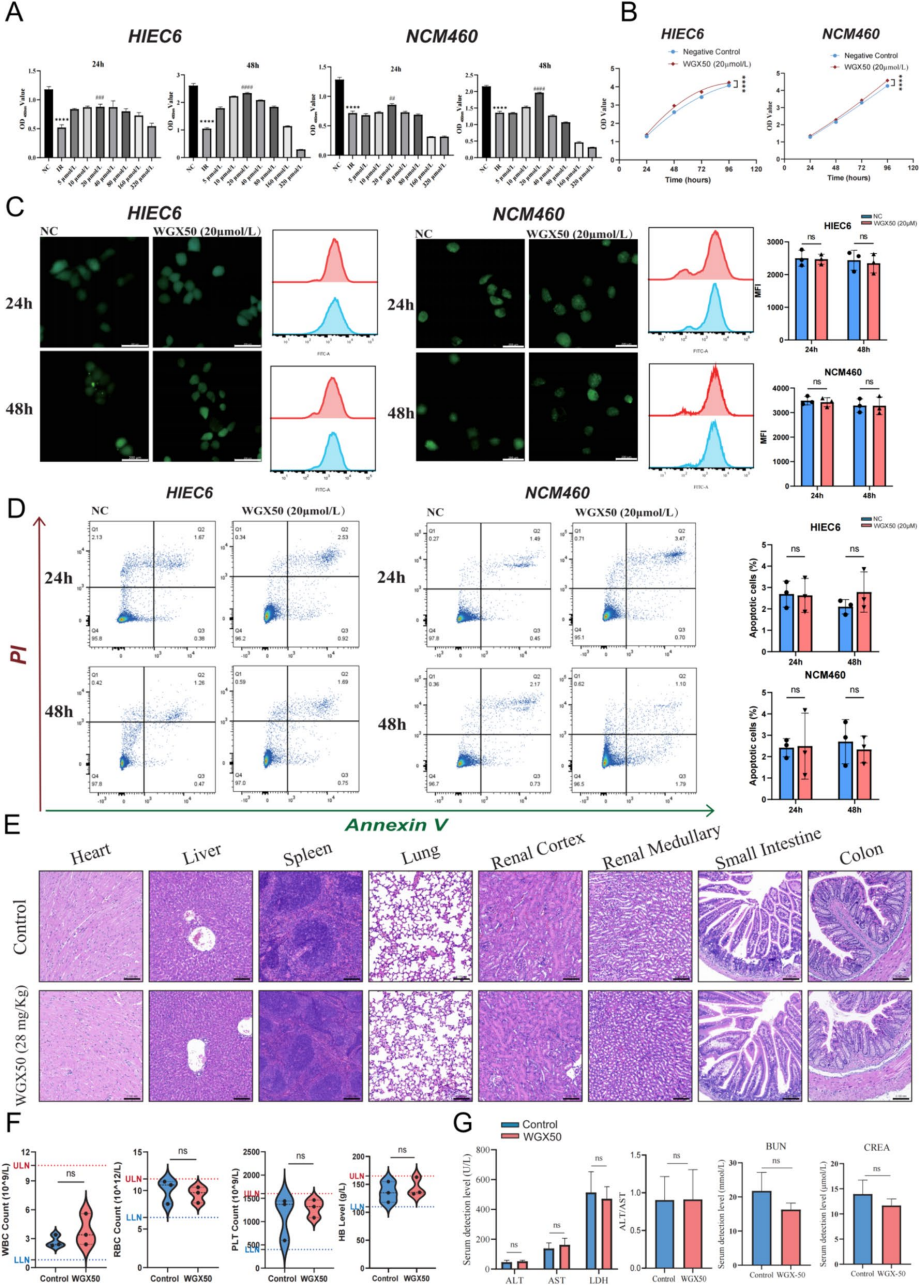
Toxicity assessment of WGX50 in vitro and in vivo. **A** Bar graph of cell proliferation rates for HIEC6 and NCM460 cells treated with different concentrations of WGX50 and irradiated with 6 Gy for 24 and 48 h (*n* = 3 per group). **B** Cell proliferation curves for HIEC6 and NCM460 cells treated with 20 μM WGX50 and negative control group at 24, 48, 72, and 96 h (*n* = 3 per group). **C** ROS immunofluorescence staining and flow cytometry analysis of HIEC6 and NCM460 cells treated with 20 μM WGX50 and negative control group at 24 and 48 h. The scale bar represents 200 μm. **D** Annexin V/PI dual-staining apoptosis analysis of HIEC6 and NCM460 cells treated with 20 μM WGX50 and negative control group at 24 and 48 h. The Q2 + Q3 quadrants represent the percentage of apoptotic cells. **E** H&E staining of heart, liver, spleen, lungs, kidneys, and intestines in C57BL/6 mice following four weeks of oral administration of 28 mg/kg WGX50 (WGX50 group) or an equal volume of saline (Control group). The scale bar represents 100 μm. **F** Comparative statistical analysis of hematologic parameters between the WGX50 and Control groups (*n* = 3 per group), including white blood cells (WBC), red blood cells (RBC), platelets (PLT), and hemoglobin (HB). **G** Biochemical analysis of liver and kidney function indicators in the WGX50 and Control groups (*n* = 3 per group), including alanine aminotransferase (ALT), aspartate aminotransferase (AST), lactate dehydrogenase (LDH), blood urea nitrogen (BUN), and creatinine (CREA). Significance: ns, no significant difference; *P < 0.05, **P < 0.01, ***P < 0.001, ****P < 0.0001; ##*P* < 0.01, ###*P* < 0.001, ####*P* < 0.0001

To explore the protective effects of WGX50 against radiation-induced intestinal cell damage, we first incubated human intestinal epithelial cells (HIEC6) and colon cells (NCM460) with different concentrations of WGX50 after radiation exposure. Cells were divided into the negative control group (NC), irradiation-only group (IR), and groups treated with varying concentrations of WGX50, followed by 6 Gy irradiation. The cell viability was assessed by CCK-8 assay at 24 and 48 h post-irradiation, measuring OD 450 nm values. The results showed that, compared to the NC group, both HIEC6 and NCM460 cell proliferation significantly decreased after 6 Gy irradiation at both 24 and 48 h. However, treatment with 20 μM WGX50 alleviated radiation-induced cell death. As the concentration of WGX50 increased, its protective effect weakened, with concentrations of 160 μM and higher significantly inhibiting cell viability (Fig. 2A). Further investigation of the cytotoxicity of 20 μM WGX50 showed that this concentration promoted the proliferation of HIEC6 and NCM460 cells (Fig. 2B). Moreover, immunofluorescence and flow cytometry analysis using DCFH-DA and CellROX probes showed no significant difference in the average ROS fluorescence intensity between the NC and 20 μM WGX50 groups (Fig. 2C). Additionally, the apoptosis rate of cells in the WGX50 group did not significantly increase compared to the NC group (Fig. 2D). These findings suggest that 20 μM is the most effective concentration for treating HIEC6 and NCM460 cells with WGX50, and at this concentration, WGX50 exhibits no noticeable cytotoxicity.

To further validate the in vivo safety of WGX50, we administered C57BL/6 mice with 28 mg/kg WGX50 by gavage daily and closely monitored their activity. After four weeks of treatment, the mice displayed normal vital signs. Histological analysis using H&E staining showed no significant morphological differences in the heart, liver, spleen, lungs, kidneys, or intestines of the WGX50-treated mice compared to the control group (Fig. 2E). Hematological and biochemical tests revealed that the WBC, RBC, PLT, and HB levels in the WGX50-treated mice were within the normal range (Fig. 2F), and there were no significant differences in ALT, AST, LDN, BUN, and CREA levels compared to the control group (Fig. 2G). Comprehensive analysis of histopathology, hematological parameters, and biochemical indicators demonstrated that long-term administration of 28 mg/kg WGX50 did not induce significant toxic reactions in mice, including body weight loss, organ damage, or abnormalities in blood/biochemical parameters. To further evaluate the in vivo safety of WGX50, we administered two doses (28 and 56 mg/kg) for six weeks. Neither dose induced significant weight loss, intestinal structural abnormalities, or abnormalities in biochemical indicators (Fig S1A-D).

In conclusion, WGX50 demonstrates no significant toxicity both in vitro and in vivo at effective concentrations, indicating a good safety profile.

### WGX50 protects against radiation-induced cell damage and intestinal injury

**Fig. 3:**
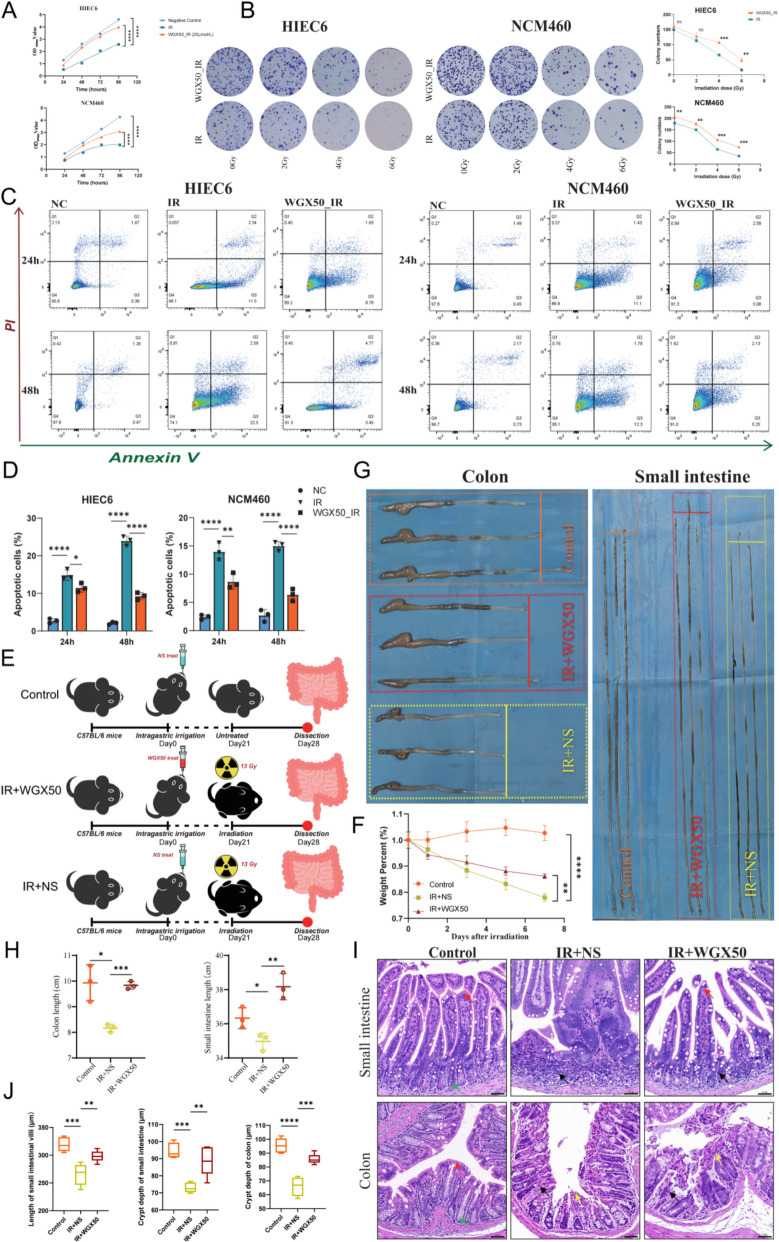
Protective effects of WGX50 on radiation-induced cell and intestinal damage. **A** Cell proliferation curves for HIEC6 and NCM460 cells in the Negative Control (Non-irradiated), IR (irradiation only), and WGX50_IR (20 μM WGX50 treatment post-irradiation) groups at 24, 48, 72, and 96 h after 6 Gy irradiation (n = 3 per group). **B** Plate colony formation images of HIEC6 and NCM460 cells in the IR and WGX50_IR groups after exposure to 0, 2, 4, and 6 Gy irradiation for two weeks. Deep blue points represent individual cell clusters, and the accompanying line graph shows the statistical analysis of colony counts. **C** Annexin V/PI dual-staining apoptosis analysis of HIEC6 and NCM460 cells in the IR and WGX50_IR groups at 24 and 48 h post-6 Gy irradiation, compared to the Negative Control group. The Q2 + Q3 quadrants represent the percentage of apoptotic cells. **D** Statistical histograms of apoptosis rates in HIEC6 and NCM460 cells from the Negative Control, IR, and WGX50_IR groups. **E** Workflow of the animal experimental protocol. **F** Weight change curves of mice in the IR + WGX50, IR + NS, and Control groups following abdominal irradiation. **G** Gross morphology images of the large and small intestines from mice in three groups. **H** Statistical analysis of the small and large intestine lengths in mice from the Control, IR + NS, and IR + WGX50 groups. **I** H&E-stained histological analysis of the small and large intestines from mice in the Control, IR + NS, and IR + WGX50 groups. The scale bar represents 50 μm. **J** Statistical comparison of villus length and crypt depth in the small intestine and crypt depth in the colon tissues from three groups. For each group, measurements were taken from 5 villi and crypts per field of view for statistical analysis. Significance: ns, no significant difference; *P < 0.05, **P < 0.01, ***P < 0.001, ****P < 0.0001

After determining the effective and safe concentrations of WGX50 for both cells and animals, we further investigated its radioprotective effects on HIEC6 and NCM460 cells as well as C57BL/6 mice. Following 6 Gy irradiation, the proliferation rate of cells in the irradiation group significantly decreased compared to the NC group, while WGX50 treatment notably alleviated the radiation-induced inhibition of proliferation in both HIEC6 and NCM460 cells (Fig. 3A). Clonogenic assay further confirmed that after 72 h of treatment with 20 μM WGX50, the number of cell colonies in both cell types significantly increased under various radiation doses compared to the IR group (Fig. 3B). Additionally, Annexin V/PI dual-staining results showed that the percentage of apoptotic cells in the WGX50_IR group at 24 and 48 h post-irradiation was significantly lower than in the IR group (Fig. 3C, D), with the reduction being more pronounced at 48 h. These results indicate that 20 μM WGX50 can significantly protect intestinal cells from radiation-induced damage.

To verify the in vivo radioprotective effect of WGX50, mice were divided into three groups: Mice in the Control group received 200 μL of saline for four weeks, while the irradiated groups were administered an equivalent volume of saline or WGX50 for three weeks before receiving abdominal radiotherapy (Fig. 3E). After a 13 Gy whole-abdomen irradiation, the mice were observed until the fourth week, after which they were euthanized and tissues collected. The weight curves showed that, compared to the Control group, the IR + NS group mice exhibited significant weight loss, while the weight loss in the IR + WGX50 group was significantly less than in the IR + NS group (Fig. 3F). Gross images revealed that at day 7 post-irradiation, the small intestine and colon of the IR + NS group mice exhibited notable shortening, thinning of the intestinal lumen, and intestinal wall atrophy, whereas the intestinal atrophy in the IR + WGX50 group mice was significantly alleviated (Fig. 3G, H). H&E staining results showed (Fig. 3I) that the Control group exhibited normal intestinal structure with abundant and well-arranged villi in the mucosal layer and intact epithelial structure (red arrows), and normal crypt structures (green arrows). The IR + NS group showed severe intestinal damage with partial erosion and shedding of the villous epithelial cells in the mucosal layer (yellow arrows), a decrease in the number of crypts, and infiltration of inflammatory cells (black arrows). The IR + WGX50 group showed significant improvement in intestinal damage compared to the IR + NS group, with intact mucosal epithelial layers, no apparent degeneration or shedding, a higher number of crypts, and reduced inflammatory cell infiltration (black arrows), although the villi arrangement was slightly less dense than in the Control group (red arrows). In addition to the histological examination, the villus length and crypt depth of small intestine, and the crypt depth of colon were measured and statistically compared across the three groups. The IR + WGX50 group exhibited notably longer villi and deeper crypts compared to the IR + NS group, indicating enhanced intestinal regeneration and protection by WGX50 (Fig. 3J). Furthermore, WGX50 pretreatment significantly improved crypt survival in both small intestine and colon tissues at 3.5 days post-irradiation (Figure S1E, F), and significantly improved the survival rate of mice after abdominal irradiation compared to the IR + NS group (Figure S1G). These results indicate that WGX50 demonstrates significant radioprotective effects both in vitro and in vivo, effectively alleviating radiation-induced cell proliferation inhibition, increased apoptosis, and intestinal tissue damage.

### WGX50 alleviates radiation-induced intestinal damage by modulating oxidative stress levels and inflammatory factors

**Fig. 4:**
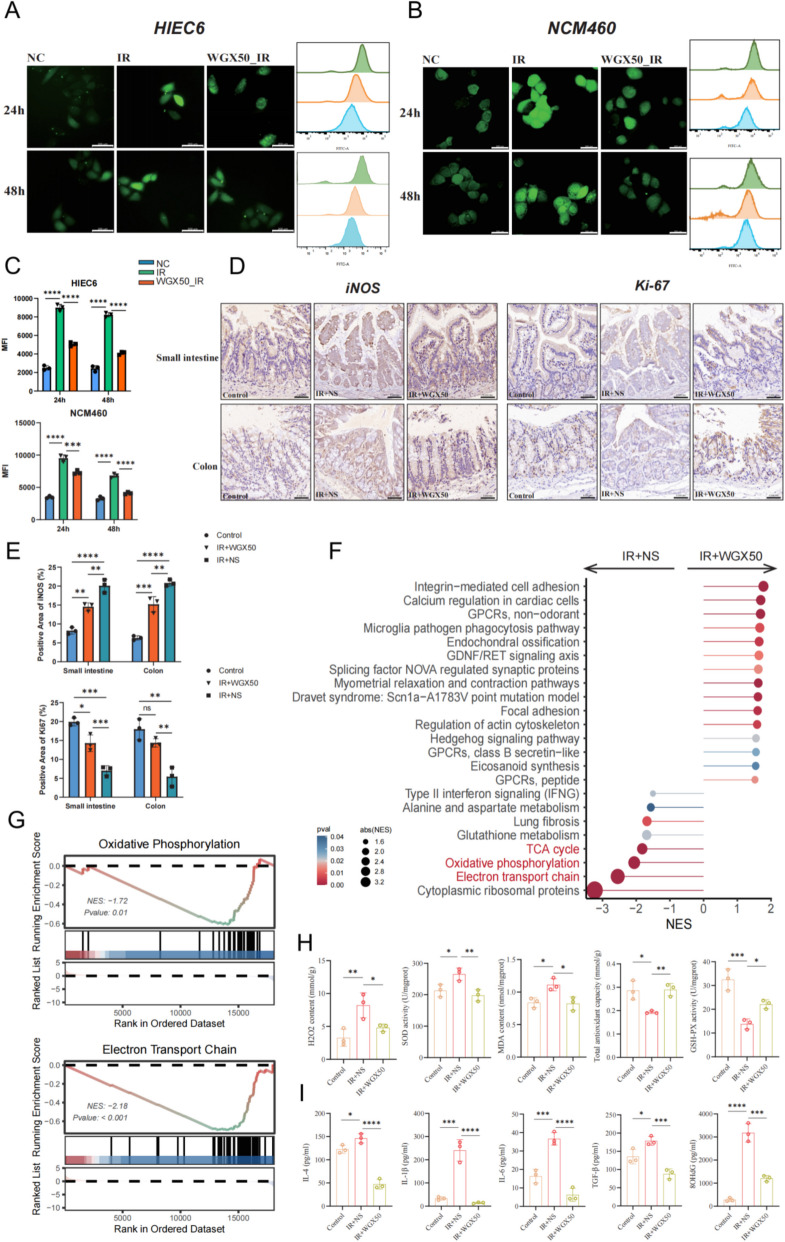
Protective effects of WGX50 against radiation-induced oxidative stress. **A**, **B** ROS immunofluorescence staining and flow cytometry analysis of HIEC6 (**A**) and NCM460 (**B**) cells in the Negative Control, IR, and WGX50_IR groups at 24 and 48 h after 6 Gy irradiation. The scale bar represents 200 μm. **C** Comparative histogram of the average fluorescence intensity of ROS from flow cytometry analysis in the three groups. **D** Immunohistochemical staining images of iNOS and Ki67 in small intestine and colon tissues from mice in the Control, IR + NS, and IR + WGX50 groups, with brown particles indicating positively stained areas for the target proteins. The scale bar represents 50 μm. **E** Statistical histogram of the relative expression of iNOS and Ki67-positive regions in the small and large intestine tissues from the three groups of mice. **F** Differential gene enrichment pathway analysis of colon tissues from IR + NS and IR + WGX50 groups of mice. **G** Down regulation of oxidative phosphorylation and electron transport chain signaling pathways in the colon tissues of IR + WGX50 group mice. **H** Statistical histogram of superoxide content in the colon tissues of mice in the Control, IR + NS, and IR + WGX50 groups. **I** Statistical histogram of inflammatory factor levels in the colon tissues of mice in the Control, IR + NS, and IR + WGX50 groups. Significance: ns, no significant difference; *P < 0.05, **P < 0.01, ***P < 0.001, ****P < 0.0001

In addition to directly damaging cellular DNA and inducing apoptosis, ROS and inflammatory factors generated by ionizing radiation are also key mechanisms of cellular damage. To explore the effects of WGX50 on cellular and intestinal oxidative stress levels, we systematically analyzed ROS levels and the overall activity of related pathways in intestinal cells and mice following irradiation. Immunofluorescence and flow cytometry detection of ROS revealed that, at 24 and 48 h post-irradiation, the oxidative stress levels in HIEC6 and NCM460 cells in the IR group were significantly higher compared to the NC group. However, WGX50 pretreatment effectively reduced ROS levels in both cell types (Fig. 4A-C). Similarly, immunohistochemical analysis of iNOS and Ki67 in the small intestine and colon tissues of the three groups of mice showed that, compared to the Control group, the IR + NS group had significantly elevated iNOS levels and decreased Ki67 expression. In contrast, the IR + WGX50 group inhibited the radiation-induced increase in iNOS and decrease in Ki67 expression (Fig. 4D, E). These results suggest that WGX50 pretreatment significantly mitigates elevated oxidative stress levels in the intestine and protects the proliferative capacity of intestinal mucosal cells.

To further investigate the molecular mechanisms, we performed transcriptomic sequencing on colon tissues from the IR + NS and IR + WGX50 groups of mice and conducted GSEA enrichment analysis using the KEGG dataset. We found that in the IR + WGX50 group, classic radiation-induced damage pathways such as the electron transport chain, oxidative phosphorylation, and the TCA cycle were significantly downregulated compared to the IR + NS group (Fig. 4F, G). Additionally, ELISA analysis of superoxide and inflammatory factors in the colon tissues of the three groups showed that, compared to the Control group, the IR + NS group exhibited a severe oxidative stress state, characterized by significantly elevated levels of oxidative products (H₂O₂, NO, and MDA) and a compensatory increase in SOD activity. However, this was accompanied by a significant reduction in total antioxidant capacity (T-AOC) and GSH-PX activity, indicating a collapse of the antioxidant defense system. In contrast, WGX50 treatment effectively mitigated oxidative stress, as evidenced by significantly lower levels of H₂O₂, NO, and MDA, a normalization of SOD activity, and a significant restoration of T-AOC and GSH-PX activity compared to the IR + NS group (Fig. 4H; Figure S1H). Furthermore, following radiation, the expression of inflammatory factors such as IL-4, IL-6, IL-1β, TGF-β, and 8-OHdG was significantly elevated, but WGX50 treatment significantly suppressed the levels of these inflammatory factors (Fig. 4I). In conclusion, WGX50 effectively mitigates radiation-induced oxidative stress and inflammatory factor elevation both in vitro and in vivo, maintaining the balance of redox reactions and thereby providing radioprotective effects.

### WGX50 protects against radiation-induced intestinal injury via ferroptosis inhibition and goblet cell preservation

**Fig. 5:**
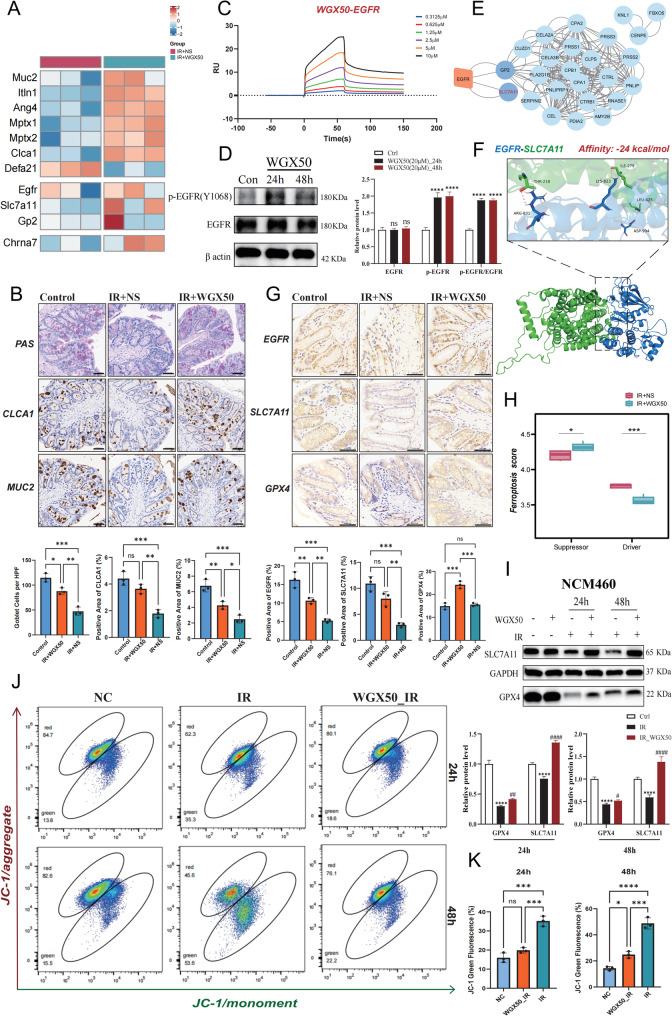
WGX50 alleviates radiation-induced intestinal damage by inhibiting ferroptosis and maintaining goblet cell function. **A** Heatmap of differential gene expression in colon tissues from IR+NS and IR+WGX50 groups of mice, with red indicating high expression of target genes and blue indicating low expression in the respective tissues (n = 3). **B** Evaluation of goblet cell quantity and morphology in colon tissues from the Control, IR+NS, and IR+WGX50 groups of mice using PAS staining and CLCA1, MUC2 immunohistochemistry, where deep purple regions in PAS staining and brown regions in immunohistochemistry indicate positive staining for goblet cells. The scale bar represents 50 μm. **C** SPR sensorgram of the binding interaction between WGX50 and human EGFR protein, with binding kinetics fitted to a 1:1 Langmuir model. **D** Western blot analysis of total EGFR and p-EGFR in NCM460 cells treated with WGX50 for 24 h and 48 h. **E** PPI network of differential genes and EGFR in colon tissues from the IR+NS and IR+WGX50 groups of mice. **F** Molecular docking image of EGFR and SLC7A11 protein-protein interaction. **G** Immunohistochemical staining and statistical histograms of the positive regions for EGFR, SLC7A11, and GPX4 in the colon tissues of Control, IR+NS, and IR+WGX50 groups of mice. The scale bar represents 50 μm. **H** ssGSEA scores for ferroptosis-related driver and suppressor genes in colon tissues from IR+NS and IR+WGX50 groups of mice.** I **Western blot analysis of the effect of irradiation and WGX50 treatment on the expression levels of SLC7A11 and GPX4 in NCM460 cells. **J **JC-1 flow cytometry analysis of mitochondrial membrane potential in NCM460 cells from NC, IR, and WGX50_IR groups. The green fluorescence indicates JC-1 monomers and mitochondrial depolarization. **K** Bar graph of the quantification of JC-1 green monomer fluorescence. Significance: ns, no significant difference; *P<0.05, **P<0.01, ***P<0.001, ****P<0.0001.

To further explore the potential mechanisms by which WGX50 effectively alleviates radiation-induced damage in the colon and rectum, we conducted a comprehensive analysis of transcriptomic data from colon tissues of two RE mouse groups. The heatmap showed that in the IR + WGX50 group, the expression of MUC2 and CLCA1 was significantly upregulated, while the expression of two repair proteins, MPTX1 and MPTX2, was also notably increased (Fig. 5A). Moreover, PAS staining and immunohistochemical analysis of MUC2 and CLCA1 revealed that the number and morphology of goblet cells in the colon of the IR + WGX50 group were significantly superior to those in the IR + NS group (Fig. 5B), suggesting that WGX50 effectively protects the structure and function of goblet cells and promotes the repair of intestinal mucosal damage. To further validate the direct interaction between WGX50 and its predicted target EGFR, we performed SPR analysis using immobilized human EGFR protein. The total immobilization level of EGFR was approximately 11,425 RU, confirming successful coupling. Sensorgram analysis revealed that WGX50 bound to EGFR in a concentration-dependent manner with clear association and dissociation phases (Fig. 5C). Kinetic analysis showed that the K_D_ of WGX50 for EGFR was 5.86 μM (5.86 × 10⁻⁶ M), with the K_a_ of 7.41 × 10^3^ (1/M·s) and K_d_ of 4.34 × 10⁻^2^ (1/s). These data indicate a strong binding affinity between WGX50 and EGFR.

To determine whether WGX50 induces sustained activation of EGFR, we treated NCM460 cells with WGX50 for 24 and 48 h and performed Western blot analysis to assess the expression of total EGFR and p-EGFR. Compared to the control group, p-EGFR levels and the p-EGFR/EGFR ratio were significantly increased following WGX50 treatment at both time points, indicating activation of EGFR signaling. However, no further increase in the p-EGFR/EGFR ratio was observed at 48 h compared to 24 h, suggesting that prolonged exposure to WGX50 does not lead to excessive EGFR hyperactivation (Fig. 5D). PPI network predictions indicated that Solute Carrier Family 7 Member 11 (SLC7A11) and Glycoprotein 2 (GP2) might be downstream molecules of EGFR. Heatmap results also showed that the expression level of EGFR in the colon tissues of the IR + WGX50 group was significantly higher than in the IR + NS group, further promoting the expression of its downstream factors, SLC7A11 and GP2 (Fig. 5A, E).

Given that SLC7A11 plays a crucial role in regulating cellular redox balance and ferroptosis, and its expression was more significantly upregulated compared to GP2 in the sequencing results, we focused on SLC7A11 for further investigation. Molecular docking results demonstrated a strong interaction between EGFR and SLC7A11, with the binding free energy of the optimal docking mode being −24.0 kcal/mol, indicating a highly stable interaction (Fig. 5F). This finding further supports the possibility that EGFR regulates SLC7A11 expression to exert radioprotective effects. SLC7A11 promotes cysteine uptake and glutathione (GSH) synthesis, maintaining cellular antioxidant capacity, while its downstream molecule, Glutathione Peroxidase 4 (GPX4), uses GSH to clear lipid peroxides and inhibit ferroptosis. Therefore, the upregulation of SLC7A11 and GPX4 likely mediates the radioprotective effects of WGX50 (Koppula et al. 2021). Additionally, the area of positive staining for EGFR, SLC7A11, and GPX4 in the colon tissues of IR + WGX50 mice was significantly higher than in the IR + NS group (Fig. 5G). The ssGSEA scores based on ferroptosis-related gene sets further confirmed that WGX50 significantly inhibited the ferroptosis levels in the colon tissues of the RE mouse model (Fig. 5H).

To validate these findings, we performed Western blot experiments in NCM460 cells. The results showed that WGX50 treatment alone did not affect the expression of SLC7A11 and GPX4, but after irradiation for 24 and 48 h, WGX50 significantly increased the expression levels of SLC7A11 and GPX4 (Fig. 5I). Furthermore, JC-1 flow cytometry analysis indicated that the mitochondrial membrane potential in the WGX50_IR group cells was significantly restored, with a significant decrease in monomeric form cells compared to the IR group (Fig. 5J, K), further confirming that WGX50 protects cellular function by inhibiting ferroptosis. Our findings suggest that WGX50 may play a potential role in alleviating radiation-induced intestinal damage by upregulating the EGFR-SLC7A11-GPX4 signaling pathway, inhibiting ferroptosis, and protecting the structure and function of goblet cells.

### WGX50 attenuates radiation-induced intestinal injury by restoring gut microbiota balance

**Fig. 6:**
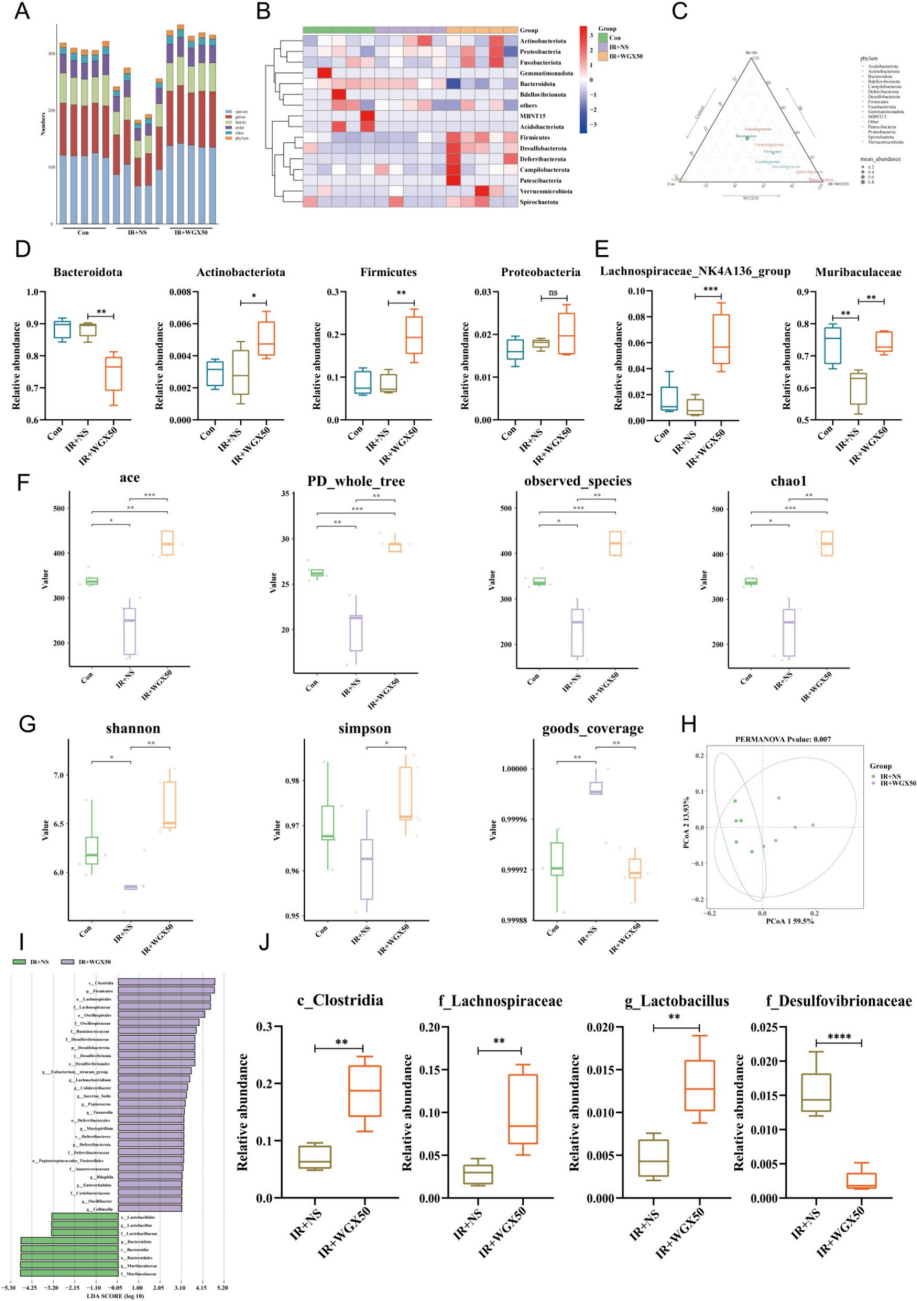
Analysis of the impact of WGX50 on gut microbiota in Control, IR + NS, and IR + WGX50 groups of mice. **A** Bar chart showing the relative abundance of gut microbiota in the three groups of mice (*n* = 5). **B** Heatmap of gut microbiota expression in the three groups, with red indicating high expression of target microbiota and blue indicating low expression. **C** Phylum-level triangular plot, where points closer to an endpoint indicate enrichment of the target microbiota in that group. **D** Bar chart of differential expression of gut microbiota at the phylum level across the three groups. **E** Bar chart of differential expression of gut microbiota at the genus level across the three groups. **F** α-diversity analysis of gut microbiota in the three groups of mice, including species richness (ACE, Chao1, Observed-species) and phylogenetic diversity (PD-whole-tree). **G** Analysis of microbiota diversity and evenness in the three groups, including Shannon index, Simpson index, and Goods-coverage value. **H** Principal Coordinate Analysis (PCoA) of gut microbiota from the IR + NS and IR + WGX50 groups. **I** LEfSe analysis showing the expression differences in gut microbiota between the IR + NS and IR + WGX50 groups. **J** Bar chart of the expression differences of ferroptosis-related microbiota in the IR + NS and IR + WGX50 groups. Significance: ns, no significant difference; *P < 0.05, **P < 0.01, ***P < 0.001, ****P < 0.0001

Finally, we collected fresh fecal samples from three groups of mice for 16S rRNA sequencing to investigate the effects of WGX50 and radiation on the gut microbiota composition. The results showed that the gut microbiota abundance in the IR + NS group was significantly reduced, indicating that abdominal irradiation significantly decreased microbiota diversity, while the microbiota diversity in the IR + WGX50 group showed no significant difference compared to the Control group (Fig. 6A). The phylum-level microbiota distribution heatmap revealed a significant upregulation of various beneficial bacterial populations in the IR + WGX50 group (Fig. 6B). The triangular plot at the phylum level further indicated significant enrichment of multiple beneficial bacterial populations in the IR + WGX50 group (Fig. 6C). Differential analysis showed that, at the phylum level, the dominant bacterial group in the IR + NS group was *Bacteroidota*, whereas in the IR + WGX50 group, the dominant groups were *Actinobacteria* and *Firmicutes* (Fig. 6D). At the genus level, the relative abundance of beneficial bacteria *Lachnospiraceae_NK4A136* and *Muribaculaceae* significantly increased in the IR + WGX50 group (Fig. 6E).

α-diversity analysis showed that the species richness (ACE, Chao1, Observed-species) and phylogenetic diversity (PD-whole-tree) of the IR + NS group were significantly lower compared to the Control and IR + WGX50 groups (Fig. 6F). The Shannon and Simpson indices in the IR + WGX50 group were higher than those in the IR + NS group, suggesting that WGX50 partially alleviated the decrease in microbiota diversity and evenness caused by radiation. Furthermore, the Goods-coverage value in the IR + NS group was significantly higher than in the Control and IR + WGX50 groups, indicating that radiation may lead to microbiota structural simplification, thereby increasing sequencing coverage (Fig. 6G). PCoA analysis showed that the sample points in the IR + NS group were more tightly clustered, further supporting the conclusion that radiation induced microbiota structural simplification, while WGX50 treatment increased the diversity of the microbiota composition (Fig. 6H).

LEFSe analysis further revealed significant differences in the relative abundance of ferroptosis-related microbiota between the IR + WGX50 and IR + NS groups. In the IR + WGX50 group, bacteria associated with the inhibition of ferroptosis, such as *C_Clostridia*, *F_Lachnospiraceae*, and *G_Lactobacillus*, were significantly upregulated, while *F_Desulfovibrionaceae*, a group associated with the promotion of ferroptosis, was significantly downregulated (Fig. 6I, J). These microbiota may indirectly influence the ferroptosis process by modulating the gut microenvironment and metabolic products, providing new insights into how WGX50 alleviates radiation-induced intestinal damage.

## Discussion

Radiation therapy is a cornerstone treatment for tumors, significantly improving patient prognosis and survival rates, either as a standalone therapy or in combination with surgery and chemotherapy. However, achieving curative outcomes often necessitates high doses of radiotherapy, which are typically delivered to tumors located near normal tissues that are sensitive to radiation. Consequently, radiation therapy inevitably causes structural damage and functional impairment in adjacent healthy tissues, resulting in both acute and chronic radiation-induced side effects (Citrin 2017). RE is one of the most prevalent complications associated with radiotherapy. It is estimated that approximately 80% of patients receiving pelvic and abdominal radiotherapy will develop acute radiation enteritis, with around 20% of these cases progressing to chronic intestinal toxicity (Loge et al. 2020). At present, treatment options for RE are limited, with no specific therapy available. Current approaches are primarily aimed at symptom management, and patients often face the risk of recurrent and persistent conditions. Therefore, there is a critical need for the development of targeted and effective therapeutic agents for the prevention and treatment of RE. WGX50 is a naturally occurring amide alkaloid, and prior research from our team has demonstrated its significant anti-inflammatory, anti-aging, and neuroprotective effects (Shi et al. 2016; Hou et al. 2017; Tai et al. 2023; Jiang et al. 2024). In this study, we found that WGX50 effectively prevents radiation-induced damage to intestinal cells and tissues in both in vitro and in vivo models, thereby significantly alleviating the symptoms of RE. These findings suggest that WGX50 holds promise as a potential new strategy for the prevention and treatment of radiation enteritis.

The safety of WGX50 was thoroughly evaluated under experimental conditions. Toxicity assessments revealed that at a concentration of 20 μM, treatment of HIEC6 and NCM460 cell lines for 24 and 48 h did not significantly elevate ROS levels or apoptosis rates, but instead promoted cell proliferation. In in vivo studies, mice were administered WGX50 at a dose of 28 mg/Kg daily for four weeks. No significant structural alterations were observed in vital organs, including the heart, liver, lungs, spleen, kidneys, and intestines. Routine biochemical parameters remained within normal limits. Furthermore, an extended six-week safety assessment at both 28 mg/kg and a higher dose of 56 mg/kg confirmed the absence of significant toxicity, as evidenced by normal biochemical indicators, organ morphology, and the absence of cardiac damage. These findings support the biological safety of WGX50, providing essential preliminary evidence for its potential application in the prevention and treatment of RE.

Radiotherapy induces cellular damage via two primary mechanisms: first, by directly interacting with biomolecules, resulting in ionization and excitation that disrupt molecular structures such as nucleic acids, proteins, and enzymes, thereby leading to direct cellular death; and second, by generating ROS through interactions with water molecules, which subsequently causes indirect cellular damage(Kusumoto et al. 2021; Monfared et al. 2023). Our research demonstrates that WGX50 significantly reduces radiation-induced apoptosis and proliferation inhibition in HIEC6 and NCM460 cells, highlighting its protective effects on intestinal epithelial cells. Additionally, after three weeks of preventive WGX50 administration, followed by whole-abdominal radiotherapy, we observed a substantial improvement in the clinical symptoms of RE model mice. These mice exhibited better overall health, with a significantly slower rate of weight loss compared to the IR + NS group. Furthermore, intestinal wall atrophy and inflammatory exudates in both the small intestine and colon were markedly reduced. Consistently, supplementary experiments showed that WGX50 pretreatment significantly increased crypt survival at 3.5 days post-irradiation, and prolonged overall survival in irradiated mice. Histological analysis revealed that, in the IR + WGX50 group, mucosal epithelial ulceration, detachment, and crypt cell necrosis were significantly less severe than in the IR + NS group. These findings suggest that WGX50 effectively protects intestinal mucosal cells from direct radiation-induced damage, thereby alleviating the pathological progression of RE. Moreover, while radiation exposure caused a significant increase in ROS levels in both cells and intestinal tissues, preventive administration of WGX50 notably reduced ROS levels in both cellular and intestinal compartments. Additionally, several oxidative stress-related signaling pathways, including oxidative phosphorylation, the TCA cycle, and the electron transport chain, were inhibited. Levels of various inflammatory factors and superoxide were also reduced. These results suggest that WGX50 mitigates oxidative stress and inflammation, further supporting its protective role in radiation-induced intestinal damage. *Zanthoxylum bungeanum Maxim*, a traditional condiment with a long history of use in both culinary and medicinal contexts, has been widely employed for its analgesic, anti-inflammatory, and antibacterial properties (Liu et al. 2021). WGX50, the active compound derived from this plant, has been previously shown by our team to possess potent antioxidant activity, providing a solid theoretical foundation for its application in radiation protection. Overall, WGX50 not only effectively prevents radiation-induced damage to the intestinal mucosa but significantly enhances crypt survival at 3.5 days post-irradiation and improves overall survival in irradiated mice. Moreover, it reduces the generation of ROS and inflammatory factors, thereby blocking oxidative stress-induced damage to intestinal tissue structure and function. This dual mechanism of action underscores its potential as a protective agent against radiation-induced intestinal injury, laying an important theoretical foundation for its use in preventing and treating RE.

Preliminary network pharmacology analysis suggested that EGFR may be a key target of WGX50 in the treatment of RE. Molecular docking, dynamic simulations and SPR analysis further confirmed that WGX50 could stably bind to EGFR. Differential gene expression analysis in two RE mouse models revealed a significant upregulation of EGFR expression in the colon tissues of IR + WGX50 mice. In addition, the Western blot analysis indicates that WGX50 does not induce excessive EGFR activation, thus confirming its role in maintaining controlled activation of EGFR signaling without overstimulation. Using PPI network analysis, we identified that the downstream target of EGFR, SLC7A11, showed the most pronounced upregulation in the IR + WGX50 group. Previous studies have demonstrated that EGFR activates the expression of SLC7A11 through downstream signaling pathways, promoting cysteine uptake and GSH synthesis, thus regulating oxidative stress and inhibiting ferroptosis (Tsuchihashi et al. 2016; Zhang et al. 2021; Wang et al. 2025). As a critical component of the system Xc-, the upregulation of SLC7A11 enhances GSH synthesis and inhibits lipid peroxidation, thereby significantly increasing cellular resistance to ferroptosis (Koppula et al. 2021; Liu et al. 2024). Additionally, ssGSEA revealed that ferroptosis activity scores in the IR + WGX50 group were significantly lower than those in the IR + NS group. Immunohistochemistry further confirmed that the protein expression levels of EGFR and SLC7A11 were significantly upregulated in the colon tissues of IR + WGX50 mice. GPX4, a key regulator of ferroptosis, is tightly associated with SLC7A11 through GSH metabolism and lipid peroxidation, forming a critical molecular axis for cellular antioxidant defense and ferroptosis regulation (Xu et al. 2023). Our results showed that, following WGX50 pretreatment, the expression of GPX4 in the colon tissues of RE mice was significantly upregulated compared to the IR + NS group. Further cellular experiments indicated that after WGX50 treatment, NCM460 colon cells displayed significantly higher levels of SLC7A11 and GPX4 expression post-radiation, and radiation-induced mitochondrial membrane potential collapse was effectively alleviated. These findings suggest that the EGFR-SLC7A11-GPX4-ferroptosis signaling axis may be a crucial molecular mechanism through which WGX50 effectively mitigates gastrointestinal radiation injury.

Furthermore, based on 16S rRNA sequencing of the intestinal microbiota, we observed that preventive administration of WGX50 resulted in a significant increase in gut microbiota richness in RE model mice compared to the NS-treated control group. Additionally, various beneficial bacterial populations, including *Lactobacillus*, *Lachnospiraceae*, and *Clostridia*, exhibited a significant increase in relative abundance, while the harmful bacteria *Desulfovibrionaceae* showed a notable decrease. These changes in the microbiota may influence the gut microenvironment, affecting iron absorption and metabolism, thereby modulating intestinal ferroptosis and inflammatory responses (Huang et al. 2023; Zhang et al. 2024; Deng et al. 2025). These findings suggest that WGX50 may alleviate radiation-induced ferroptosis in intestinal tissues by regulating the abundance of specific gut microbiota, thereby enhancing its protective effects on the intestine.

While our research demonstrates that WGX50 significantly protects against radiation-induced intestinal mucosal damage, there are some limitations to consider. First, the long-term safety and optimal dosing regimen of WGX50 have not been fully established. Future studies are needed to assess its safety and pharmacokinetics across different doses and administration routes to optimize its clinical application. Second, the mechanism by which WGX50 prevents RE through the inhibition of ferroptosis is primarily based on colon tissue in animal models, and similar effects have not been observed in small intestine tissues. This discrepancy may be due to the use of a single high-dose irradiation model in this study, as the small intestine, being more mobile during radiotherapy, may not exhibit the same pronounced phenotypic differences as the more fixed large intestine. Finally, while 16S rRNA sequencing revealed that WGX50 significantly modulates the gut microbiota structure, the precise mechanisms underlying these changes and their role in RE prevention require further investigation. Therefore, future studies should focus on refining RE animal models and exploring segment-specific differences in signaling pathway expression to fully elucidate the mechanisms by which WGX50 protects the gut from radiation-induced damage.

## Conclusions

WGX50 significantly mitigates radiation-induced intestinal mucosal ulceration and inflammatory infiltration in mice by inhibiting apoptosis and oxidative stress in intestinal epithelial cells, thereby effectively reducing the toxicity of radiotherapy. Furthermore, it may regulate the EGFR-SLC7A11-GPX4 signaling axis, thereby inhibiting radiation-induced ferroptosis in intestinal mucosal cells and protecting normal intestinal tissues from radiation damage. Based on these findings, we propose that WGX50 holds great potential as a therapeutic agent for the prevention and treatment of radiation-induced intestinal injury.

## Supplementary Information

Supplementary Material 1.

## Data Availability

No datasets were generated or analysed during the current study.

## Abbreviations

Aβ: Amyloid-β oligomers
AD: Alzheimer’s disease
ALT: Alanine aminotransferase
AST: Aspartate aminotransferase
BUN: Blood urea nitrogen
CASP3: Cysteine-dependent aspartate-specific protease-3
cDNA: Complementary DNA
CK: Creatine kinase
CTnT: Cardiac troponin T
CREA: Creatinine
DOX: Doxorubicin
EGFR: Epidermal Growth Factor Receptor
GSH: Glutathione
GP2: Glycoprotein 2
GPX4: Glutathione peroxidase 4
HB: Hemoglobin
IMRT: Intensity-modulated radiotherapy
K_a_: Calculate association
K_d_: Dissociation
K_D_: Equilibrium dissociation constants
LDH: Lactate dehydrogenase
MCC: Maximal Clique Centrality
MOE: Molecular Operating Environment
NO: Nitric oxide
NSAIDs: Non-steroidal anti-inflammatory drugs
PBI: Partial-body irradiation
PCR: Polymerase chain reaction
PI: Propidium iodide
PCoA: Principal Coordinate Analysis
PME: Particle Mesh Ewald
PPI: Protein-protein Interaction
RBC: Red blood cell count
RE: Radiation enteritis
Rg: Radius of gyration
RMSD: Root mean square deviation
ROS: Reactive oxygen species
SLC7A11: Solute Carrier Family 7 Member 11
SSD: Source-to-skin distance
SPR: Surface plasmon resonance
WBC: White blood cell count
PLT: Platelet count
