# Supplementary material for: WGX50 attenuates radiation enteritis by targeting ferroptosis and redox homeostasis via EGFR
Source: Mol Med. 2025 Oct 8;31:309. doi: 10.1186/s10020-025-01375-3 (PMC12505694; doi:10.1186/s10020-025-01375-3)
Supplement: Supplementary file 1 — Supplementary Material 1. [file 10020_2025_1375_MOESM1_ESM.docx]

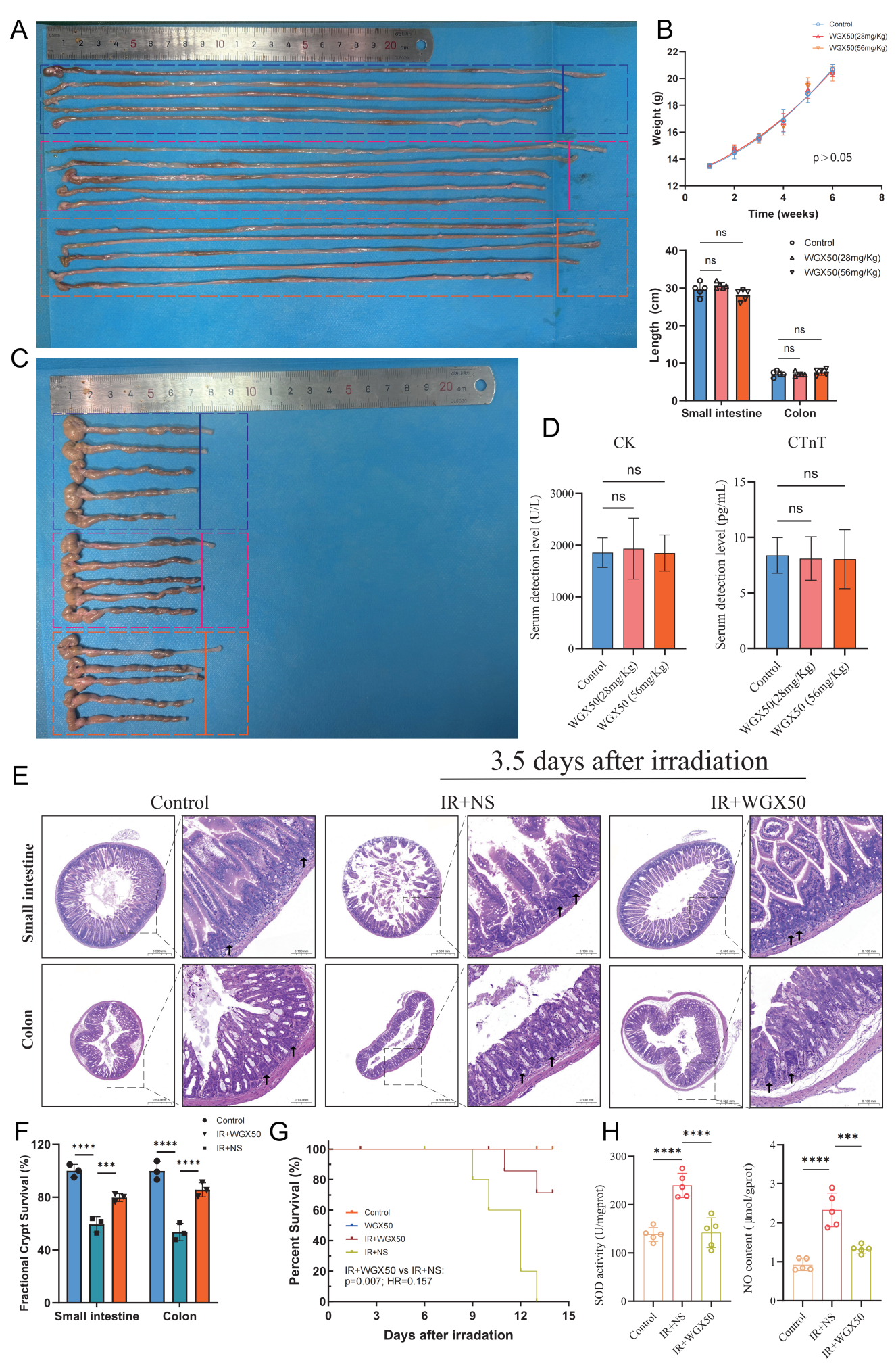


**Fig S1** Supplementary in vivo experimental data of WGX50. (A) Gross images of the small intestine from Control, WGX50 (28 mg/kg), and WGX50 (56 mg/kg) groups after six weeks of gavage (n=5). (B) Body weight curves of mice during gavage across the three groups. (C) Gross images of the colon and statistical analyses of small intestine and colon lengths from Control, WGX50 (28 mg/kg), and WGX50 (56 mg/kg) groups after six weeks of gavage. (D) Serum levels of creatine kinase (CK) and cardiac troponin T (cTnT) in the three groups after six weeks of gavage. (E) H&E staining of small intestine and colon collected 3.5 days post-abdominal irradiation from Control (non-irradiated), IR+NS, and IR+WGX50 groups. Representative fields at 4.5× and 20× magnifications are shown. Black arrows indicate surviving crypts with ≥10 basophilic epithelial cells, intact structure, and distinct mitotic figures (n = 3). (F) Quantification of crypt survival rate based on (E). (G) Survival curves of Control, WGX50, IR+NS, and IR+WGX50 group mice after abdominal irradiation (n=5). (H) ELISA analysis of superoxide dismutase (SOD) activity and nitric oxide (NO) content in colonic tissues from Control, IR+NS, and IR+WGX50 groups. Significance: ns, not significant; *P < 0.05, **P < 0.01, ***P < 0.001, ****P < 0.0001.
